# The Genomic Landscape of a Restricted ALL Cohort from Patients Residing on the U.S./Mexico Border

**DOI:** 10.3390/ijerph18147345

**Published:** 2021-07-09

**Authors:** Alice Hernandez Grant, Yoshira Marie Ayala-Marin, Jonathon Edward Mohl, Elisa Robles-Escajeda, Georgialina Rodriguez, Julie Dutil, Robert Arthur Kirken

**Affiliations:** 1 Department of Biological Sciences, College of Science, The University of Texas at El Paso, El Paso, TX 79968, USA; aehernandez6@utep.edu (A.H.G.); ymayala2@utep.edu (Y.M.A.-M.); erobles3@utep.edu (E.R.-E.); grodriguez@utep.edu (G.R.); 2 Department of Mathematical Sciences, College of Science, The University of Texas at El Paso, El Paso, TX 79968, USA; jemohl@utep.edu; 3 Department of Biochemistry, Cancer Biology Division, Ponce Research Institute, Ponce Health Sciences University, Ponce, PR 00716, USA; jdutil@psm.edu

**Keywords:** ALL, relapse, precision medicine, Hispanic health disparities, *KRAS*, *STAT5B*, *NOTCH2*, *ROS1*, *WT1*

## Abstract

Next-generation sequencing (NGS) has identified unique biomarkers yielding new strategies in precision medicine for the treatment of Acute lymphoblastic leukemia (ALL). Hispanics show marked health disparities in ALL, often absent in clinical trials or cancer research. Thus, it is unknown whether Hispanics would benefit equally from curated data currently guiding precision oncology. Using whole-exome sequencing, nine ALL patients were screened for mutations within genes known to possess diagnostic, prognostic and therapeutic value. Genes mutated in Hispanic ALL patients from the borderland were mined for potentially pathogenic variants within clinically relevant genes. *KRAS* G12A was detected in this unique cohort and its frequency in Hispanics from the TARGET-ALL Phase II database was three-fold greater than that of non-Hispanics. *STAT5B* N642H was also detected with low frequency in Hispanic and non-Hispanic individuals within TARGET. Its detection within this small cohort may reflect a common event in this demographic. Such variants occurring in the MAPK and JAK/STAT pathways may be contributing to Hispanic health disparities in ALL. Notable variants in *ROS1*, *WT1*, and *NOTCH2* were observed in the ALL borderland cohort, with *NOTCH2* C19W occurring most frequently. Further investigations on the pathogenicity of these variants are needed to assess their relevance in ALL.

## 1. Introduction

Acute lymphoblastic leukemia (ALL) is a malignant transformation of lymphoid precursor cells commonly occurring in children. Frontline therapies, typically multi-agent chemotherapy strategies, have led to remission in 95% of pediatric ALL. However, a subgroup does exhibit refractory or ALL relapse, and the five-year free survival rate is reduced to 15–50% [1]. This subgroup is disproportionately reported for Hispanic children who experience a 5% lower five-year free survival rate for ALL than their non-Hispanic counterparts [2]. The etiology for ALL relapse is unclear, but advances in stratification based on chromosomal abnormalities and genetic alterations are guiding their prognosis and treatment management [3]. These improvements in stratification are largely derived from next-generation sequencing (NGS) studies that have revealed unique biomarkers associated with diagnostic criteria as well as prognostic relevance of leukemias, yielding to the revised 2016 classifications of acute leukemias by the World Health Organization [4]. These classifications involve chromosomal translocations/fusions that are common in ALL; while others such as Ph-like ALL that are negative for example the BCR–ABL1 fusion rely on the detection of kinase-activating aberrations, including rearrangements, mutations, and copy number alterations [5]. In other ALL subtypes, these are observed as co-operating secondary aberrations [6]. In acute myeloid leukemia (AML), genetic aberrations have practical value in treating AML [7] and similarly, ALL genetic aberrations detected by NGS [8] have improved ALL treatment. NGS will continue to influence the diagnosis and prognosis of acute leukemias as clinical oncology progresses toward precision medicine. These efforts are especially needed for improving treatment regimens for refractory and relapse leukemias, including ALL.

It is unknown whether Hispanic patients benefit equally from precision oncology due to a lack of inclusion or participation of minorities in cancer research [9,10]. For example, in Texas, estimates of less than 2% of genome-wide studies on biospecimens are obtained from Hispanics [11]. Therefore, well-known cancer mutations that are principally derived from large databases may or may not reflect what is observed in under-represented Mexican-American Hispanic cancer patients. Furthermore, it is unknown whether clinically relevant genes are responsible for driving ALL in this population. To explore the mutational landscape involved in ALL, we performed NGS on ALL patients from the Paso del Norte region with a demographic of 83% Hispanic, 96% of Mexican origin. This region is associated with a higher incidence of ALL and inferior survival in Hispanics [12]. Using whole-exome sequencing (WES), the genetic profile of nine ALL patients (four new-onset and five relapse cases) was investigated for mutations within genes having diagnostic, prognostic, and therapeutic value [13]. Next, genes mutated in ALL patients were mined for variants with pathogenic potential [14]. The resulting genes were then screened for their presence across ALL targetable pathways and their occurrence in the Therapeutically Applicable Research to Generate Effective Treatments (TARGET)-ALL Phase II database [15]. The recognized pathogenic variants, *KRAS* G12A and *STAT5B* N642H were detected in the ALL borderland cohort. Perhaps, alterations in the mitogen-activated protein kinase (MAPK) and the Janus kinase/signal transducer and activator of transcription (JAK/STAT) pathways are contributing to Hispanic health disparities in ALL. The *NOTCH2* C19W variation was a frequent event in the ALL borderland cohort, yet it was not present in the TARGET-ALL Phase II database. The occurrence of this variant in a biomarker gene, along with others mentioned herein, warrant investigation of their contribution to oncogenesis, given their potential relevance in clinical applications. Further NGS investigations in diverse and geographically distinct groups burdened by high incidence, prevalence, and mortality may yield novel variants driving ALL. These efforts have the potential to contribute to guidelines for the stratification of disease and improved treatment.

## 2. Materials and Methods

### 2.1. Participants and Sequencing

All research utilizing human subjects was approved by The University of Texas at El Paso Institutional Review Board (UTEP IRB) committee, and all participants provided written informed consent. Genomic DNA was collected using Puregene Kit A (Qiagen, Germantown, MD, USA) according to the manufacturer’s instructions from a small cohort of 7 healthy controls and 9 patients diagnosed with ALL in the El Paso, Texas region. Purified DNA was sent to the Otogenetics Corp. in Atlanta, GA, USA, for WES.

### 2.2. Bioinformatics

Sequences were quality trimmed, mapped to the Gchr38.p1 human reference genome, and genotyped using process_sequences.py (https://github.com/jonmohl/PopGen) (accessed on 1 April 2021) [16,17,18,19]. Analysis of the aligned WES data was performed by the Border Biomedical Research Core (BBRC) Bioinformatics unit using OncoMiner [20] to identify non-synonymous amino acid (AA) mutations [21]. Categorization of variants into germline and somatic was not possible due to the absence of matched healthy tissues. To overcome this limitation, single-nucleotide polymorphisms (SNPs) found within coding regions and not present within control samples were extracted and further analyzed. The remaining SNPs were then filtered using the GnomAD database in which any position that had a presence of more than 5% in the Latino/Admixed American group was additionally removed. This somewhat liberal approach was meant to include mostly somatic mutations while enabling the detection of rare germline mutations that may be relevant to cancer in this population. The total number of mutations per sample was compiled. Filtering and summary steps were performed using in-house Python3 scripts. Data compiled from Otogenetics (Gchr37), filtered for SNPs from select genes (96), can be downloaded from the UTEP Bioinformatics Repository (https://datarepo.bioinformatics.utep.edu/getdata?acc=B4U5YED0W0RIE3C) (accessed on 7 July 2021). The ethnicity of the ALL patient samples was determined by analysis of the germline variants obtained from WES data. The WES vcf files were intersected with dbSNP common variants (dbSNP138) [22] and mapped in reference to 1000 Genomes [23] populations using the first 2 dimensions of multidimensional scaling analysis [24]. The control participants self-identified as Hispanic (5) and non-Hispanic (2).

### 2.3. Gene Inclusion and Classification of Mutations

Gene lists with diagnostic, prognostic, and therapeutic value were obtained from OncoKB [13]. OncoKB categorizes genes by diagnostic, prognostic, and therapeutic levels of evidence [13]. Levels 1–4 are defined as follows: an FDA-recognized biomarker (Level 1), standard care biomarker (Level 2), compelling clinical evidence biomarker (Level 3), and compelling biological evidence biomarker (Level 4), where all are found with slight variation across each category. For simplicity, Levels 1–4 are referred to across diagnostic and prognostic evidence, although in OncoKB, this terminology is reserved for therapeutic indications. Therapeutic biomarkers have additional levels that are predictive of drug resistance. From this list of potentially actionable genes, those observed in new-onset and relapse ALL patients were further screened for pathogenic mutations using the Cancer Mutation Census (CMC) within the Catalogue of Somatic Mutations in Cancer (COSMIC) database [14]. Mutations found in the ALL cohort were classified according to their mutational significance in cancer ranked by tier. The CMC ranks the AA variations in three tiers, depending on the evidence supporting their role in cancer pathogenesis. Three factors that affect their rank include (1) recurrent mutations in Census (CGC) oncogenes or tumor suppressors (nonsense, large indel, and frameshift), (2) pathogenic status in Clinvar, and (3) evidence of positive selection determined by the dN/dS ratio. Tier 1 mutations meet factors 1 and 2. Tier 2 mutations have a combination of any two factors 1, 2 or 3. Tier 3 meets at least one of the factors. All other mutations in CGC are ranked as other. Here, we included an additional rank referred to as Tier X, including variants that occur in AA locations that correspond with Tier 1, Tier 2, and Tier 3; yet, the change in AA is not reported in the COSMIC database or labeled as other. Finally, genes containing Tier 1, Tier 2, Tier 3, or Tier X mutations were searched for in targetable pathways, as referenced in My Cancer Genome [15]. All relevant variants were searched against TARGET-ALL Phase II (phs000218) vcf files from white non-Hispanic (408) and white Hispanic individuals (145) to assess their frequency in a larger cohort.

## 3. Results

### 3.1. Patient Demographics

To explore the mutational landscape of Hispanic ALL patients, WES was performed on nine ALL patients from the borderland of the Paso Del Norte region. Patient demographics are shown for new-onset or relapsed ALL patients (Table 1) diagnosed with either T or B cell lineage subtypes.

### 3.2. The Genomic Landscape of ALL

Using WES, the gene mutation profile of the nine ALL patients was investigated for mutations within clinically relevant genes, including those with diagnostic, prognostic, and therapeutic value. This list of clinically relevant genes (Supplementary Figure S1) was further screened for potential variants with mutational significance. Each variant was ranked by a tier indicative of mutational significance, and, hence, potential cancer pathogenicity. Tier 1 has the highest level of mutational significance and is considered pathogenic in cancer. Tier 2 and Tier 3 have some level of mutational significance and are likely pathogenic in cancer. Tier X is suspected of having mutational significance; its contribution to cancer is unknown. Each variant was subsequently categorized by its level of clinical relevance corresponding to the particular gene.

#### 3.2.1. ALL Borderland Patients Harbor Tier Mutations Detected in First-Level Cancer Biomarkers with Therapeutic Potential

**Figure 1:**
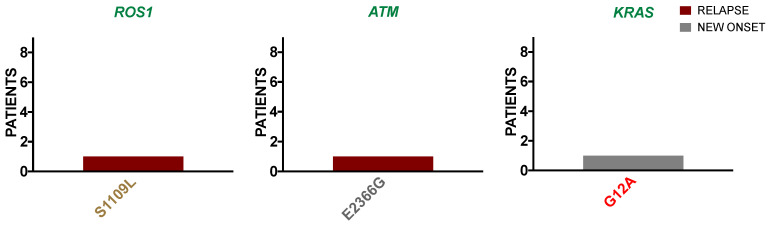
ALL borderland patients harbor tier mutations detected in first level cancer biomarkers with therapeutic potential. Variants are shown occurring in new-onset (grey) and relapse (maroon) ALL patients where mutational significance is indicated by Tier 1 (red), Tier 3 (gold), and Tier X (grey) within Level 1 clinically relevant genes (green) including *ROS1*, *ATM*, and *KRAS*.

Detected from the ALL borderland cohort were *ROS1*, *ATM*, and *KRAS*; genes with the highest level of clinical significance that were found harboring variants with mutational significance in Tier 3, Tier X, and Tier 1, respectively (Figure 1). These genes are FDA-recognized biomarkers with therapeutic indications in various cancers. Although such indications are not yet applied to ALL, mutations in these genes can contribute to hematological malignancies. For example, *ROS1* is a receptor tyrosine kinase (RTK) that can translocate to form oncogenic fusions or express activating aberrations in some hematologic malignancies [25,26]. It is unknown if any ALL patients from our cohort contained fusions in *ROS1*. The DNA damage response gene *ATM* is inactivated in various hematological malignancies [27]. A similar variant (*ATM* E2366 *) can propagate mutations associated with predispositions to cancer [28]. Lastly, mutations in the GTPase *KRAS* are prevalent in ALL and are associated with resistance and inferior survival [29]. The mutation frequency of the *KRAS* hotspot at position 12 is commonly seen in ALL relapse [30].

#### 3.2.2. ALL Borderland Patients Harbor Tier Mutations Detected in Second-Level Biomarkers with Diagnostic and Prognostic Value for Hematologic Malignancies

**Figure 2:**
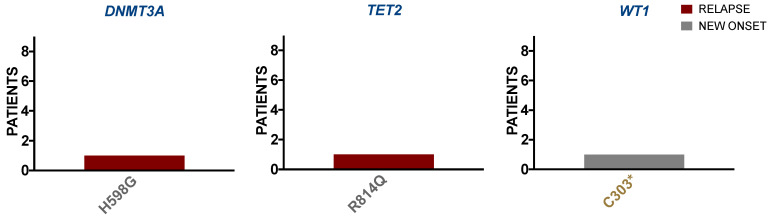
ALL borderland patients harbor tier mutations detected in second-level biomarkers with diagnostic and prognostic value for hematologic malignancies. Variants are shown occurring in new-onset (grey) and relapse (maroon) ALL patients where mutational significance is indicated by Tier X (grey) and Tier 3 (gold) within Level 2 clinically relevant genes (blue) including *DNMT3A*, *TET2*, and *WT1* involved in epigenetic regulation.

Detected in the ALL borderland cohort were genes involved in epigenetic regulation at the next level of clinical significance, including *DNMT3A*, *TET2*, and *WT1*. These genes were observed harboring variants with mutational significance in Tier X and Tier 3, respectively (Figure 2). At this second level, *DNMT3A*, *TET2*, and *WT1* are FDA-recognized biomarkers supporting diagnostic and prognostic indications in hematologic malignancies, excluding ALL. However, lower-level indications for these biomarkers are applicable to ALL prognoses. For example, *DNMT3A* mutations are associated with relapse and inferior survival outcomes in ALL [31]. Similarly, *TET2* and *WT1* aberrations are associated with poor prognosis in AML [32,33] and perhaps ALL [34,35]. The associated outcomes are partly due to the loss or interference of epigenetic regulation [36].

#### 3.2.3. ALL Borderland Patients Harbor Tier Mutations Detected in Third-Level Biomarkers with Diagnostic Value for Leukemia

**Figure 3:**
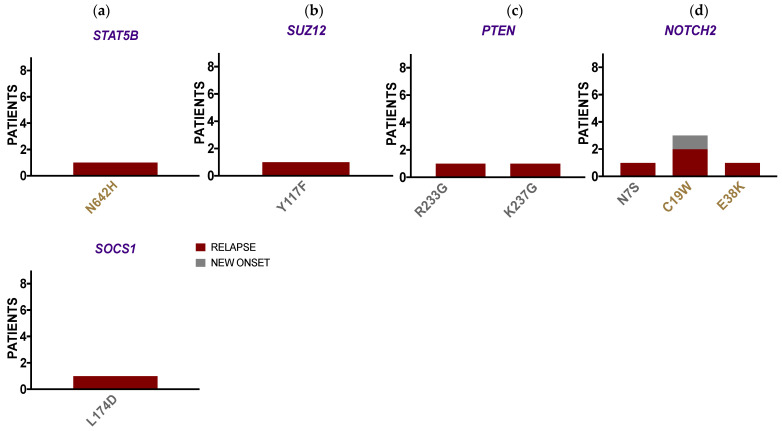
ALL borderland patients harbor tier mutations detected in third-level biomarkers with diagnostic value for leukemia. ALL mutations occur in (**a**) JAK/STAT, (**b**) epigenetic, (**c**) PI3K, and (**d**) NOTCH2 signaling genes that are used as biomarkers for the diagnosis of leukemias and other hematopoietic malignancies. Tier 3 (gold) and Tier X (grey) mutations are shown occurring in new-onset (grey) and relapse (maroon) ALL patients (superimposed bars) within Level 3 clinically relevant genes (purple).

Detected from the ALL borderland cohort were *STAT5B*, *SUZ12*, *PTEN*, *NOTCH2*, and *SOCS1*; genes at the lowest level of clinical significance, that were found harboring variants with mutational significance (Figure 3). Variants in *STAT5B* and *SOCS1* represent alterations in the JAK/STAT pathway (Figure 3a). The *SUZ12* variant represents another alteration in epigenetic genes (Figure 3b), as demonstrated in Figure 2. The *PTEN* variations represent alterations in the PI3K signaling pathway (Figure 3c). Lastly, the enrichment and frequency of *NOTCH2* variants in this ALL cohort indicated aberrations in *NOTCH2* signaling (Figure 3d). *STAT5B* and *SUZ12* at this level hold diagnostic value for T cell leukemias, while the remaining are relevant to other hematologic malignancies. There is evidence to support a role for these genes in ALL. For example, the activating *STAT5B* N642H mutation found in T cell ALL has been associated with risk of relapse [37]. *SUZ12* has been considered a tumor suppressor gene mutated in T-ALL [38]. Similarly, *PTEN* and *NOTCH2* mutations have been reported in T-ALL cases [39,40]. Lastly, changes in *SOCS1* expression has been reported in refractory AML [41].

#### 3.2.4. Prevalence of Borderland Tier Mutations in Hispanic and Non-Hispanic ALL Patients from the TARGET-ALL Phase II Database

**Figure 4:**
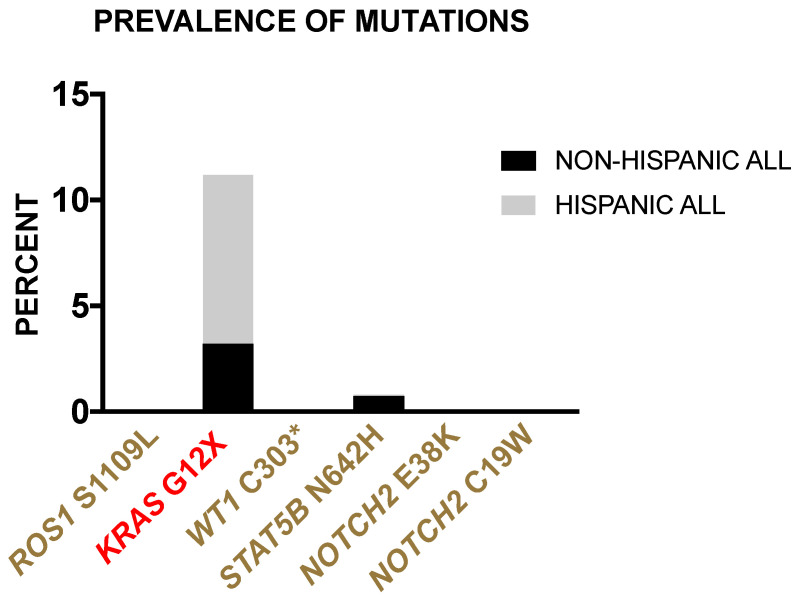
Prevalence of borderland tier mutations in Hispanic and non-Hispanic ALL patients from the TARGET-ALL Phase II database. Variants classified as Tier 1 (red) and Tier 3 (gold) are shown with their frequency in percent (superimposed bars) across Hispanic ALL patients (light gray) and non-Hispanic patients (black) from the TARGET-ALL Phase II database.

The variants at each level of clinically relevant genes were found across patients. Patient 1 harbored the *ATM*, *DNMT3A*, *TET2*, and *STAT5B* mutations. Others were individual cases including the mutation in *SUZ12* in Patient 2, *WT1* in Patient 3, *SOCS1* in Patient 5, *KRAS* in Patient 8 and the *ROS1* and *PTEN* mutations in Patient 7. *NOTCH2* mutations were dispersed, where Patient 1 contained *NOTCH2* N7S and E38K, while Patients 2, 4, 7, 8, and 9 harbored *NOTCH2* C19W. Among the select variants reported across clinically relevant genes, those classified as Tier 1–3 are the most plausible in contributing to leukemogenesis in the ALL borderland cohort. Tier X variants require exploratory investigations for oncogenic potential, if not solely for their immediate relevance to clinical application upon any discoveries. Thus, prioritizing the analysis of Tier 1–3 variants in the ALL borderland cohort, *ROS1* S1109L, *KRAS* G12A, *WT1* C303 *, *STAT5B* N642H, *NOTCH2* E38K, and *NOTCH2* C19W were screened for in the TARGET-ALL Phase II database to investigate their presence across a larger cohort. *ROS1* S1109L, *WT1* C303 *, and *NOTCH2* (C19W, E38K) mutations were not found in the larger cohort, and all occurred as individual cases in the borderland ALL cohort, except for *NOTCH2* C19W, which occurred in five of nine patients. *KRAS* G12A and *STAT5B* N642H were detected in the TARGET-ALL Phase II database. The frequency of these variants was converted to percent and compared between Hispanic and non-Hispanic white ALL patients (Figure 4). It is worth mentioning that the exact AA change in the borderland cohort was *KRAS* G12A, while the TARGET-ALL Phase II database included multiple AA changes in *KRAS* at position 12 (*KRAS* G12X). The frequency of *KRAS* G12X in the larger Hispanic cohort was roughly 11% in contrast to 3% in non-Hispanic white patients. *KRAS* G12A occurred in one of nine cases from the ALL borderland cohort, reflecting observations in the database. In contrast, the frequency of *STAT5B* N642H was similar, at roughly 8% in both Hispanic and non-Hispanic ALL patients. The *STAT5B* N642H mutation occurred in one of nine cases in the ALL borderland cohort. The detection of this somewhat rare mutation within this small borderland cohort may suggest that further sampling would reveal a more frequent event of this aberration in Hispanic ALL patients, and perhaps those of Mexican-American origin.

## 4. Discussion

The genetic diversity of ALL represents a significant challenge for precision medicine. Here, we explored the mutational landscape of ALL patients from a demographic burdened with leukemia health disparities residing in the borderland. Notable variants were identified within common cancer biomarkers. Specifically, ALL cases contained mutations in potentially actionable genes, including *KRAS* G12A and *ROS1* S1109. *KRAS* aberrations are an inclusion criterion for ongoing clinical trials in ALL (https://www.mycancergenome.org/content/gene/kras/) (accessed on 07 July 2021). *ROS1* S1109L is observed in 0.016% of the Latino/Admixed American group of the GnomAD database and is classified as a Tier 3 mutation in the COSMIC database. This mutation should be explored for its influence as a predisposing factor in cancer. Another notable variant was *WT1* C303 *. This gene is recognized as a biomarker with diagnostic value for AML; whether this variant can provide diagnostic value in ALL should be explored. *NOTCH2* mutations were frequently observed in the ALL borderland cohort as seen in T-ALL [40]. The *NOTCH2* E38K variant was also reported in B-ALL [42]. The *NOTCH2* C19W mutation has been reported in angioimmunoblastic T cell lymphoma [43]. This mutation was detected in both new-onset and relapse cases, and it seems plausible that it may be retained during the progression of ALL and contribute to relapse. An obvious shortcoming is the absence of diagnosis-relapse pair controls; thus, there is no direct verification of the identified variants as somatic. *NOTCH2* mutations have diagnostic and prognostic value for certain lymphomas that are predictive of adverse outcomes [44]. Thus, the outcomes of ALL patients harboring *NOTCH2* C19W or E38K should be explored. Lastly, the *STAT5B* N642H variant that confers poor prognosis in T-ALL [37] was also detected in our ALL cohort. Each of these variants was screened against ALL cases within the TARGET-ALL Phase II dataset, which includes a representation of 17% Hispanics. The *KRAS* G12X and *STAT5B* N642H were the only Tier 1–3 variants detected in this database. Interestingly, the occurrence of *KRAS* G12X was similar across Hispanics in both the ALL borderland cohort and the larger database, and three-fold greater than that observed in non-Hispanic whites. The occurrence of *STAT5B* N642H is likely greater in Hispanic ALL patients from the borderland compared to those within the large database. This inconsistency across datasets may be in part due to the mixed lineage termed Hispanic in the TARGET-ALL Phase II dataset, whereas the Paso del Norte borderland dataset includes Hispanics from primarily Mexican-American origin. Thus, aberrations in the MAPK pathway may be contributing to Hispanic health disparities in ALL, while aberrations in the JAK/STAT pathway may be more relevant to health disparities unique to Mexican-Americans or the borderland. An adequate number of clinical samples from diverse lineages are needed to confirm this interpretation.

In our cohort, we detected the activating *STAT5B* N642H mutation and *SOCS1* L174D that signal in the JAK/STAT pathway. It is important to note that these genes within the JAK/STAT pathway are considered biomarkers within the lowest category of clinical evidence. It is proposed that frequent sampling of diverse ALL patients may change the status of JAK/STAT genes into a higher level of clinical evidence that would be complimented by the targetability of this pathway. In B-ALL subtypes that lack known fusion transcripts, aberrant mutations within the MAPK/RTK and JAK/STAT pathways are targeted [45]. Our cohort may have been diagnosed with similar ALL subtypes, as increased frequency of Ph-like ALL is observed in Hispanics and associated with inferior outcomes [46]. Many of these subtypes are complemented by NGS data to help stratify patients and treatment regimens. Thus, the Hispanic Mexican-origin demographic that experience health disparities in ALL may benefit from NGS efforts in stratification to detect relevant biomarkers that may be overlooked in immunophenotyping and cytogenetics alone. Another pathway harboring mutated genes in our borderland ALL cohort is the chromatin remodeling pathway, where *DNMT3A*, *TET2*, *WT1*, and *SUZ12* all influence epigenetic regulation. Epigenetic modulators are frequently mutated in ALL relapse [47,48]. Given the potential to therapeutically target this pathway, tier variants observed in these genes should be further explored for their oncogenic potential in ALL. Collectively, this implies that more work is needed across a diverse population and within high-risk groups to determine new biomarkers and strategies for actionable mutations in ALL.

## 5. Conclusions

Collectively, curated data, largely derived from NGS studies, have aided in providing diagnostic, prognostic, and therapeutic biomarkers for ALL. One major finding included the detection of *KRAS* G12A and *STAT5B* N642H in Hispanic ALL borderland patients. Interestingly, the occurrence of *KRAS* G12A is similar across Hispanics in both the ALL borderland cohort and the TARGET-ALL Phase II dataset, yet greater than that observed in non-Hispanic whites. The occurrence of *STAT5B* N642H might be greater in Hispanic ALL patients from the borderland compared to those within the database. Thus, aberrations in the MAPK pathway may be contributing to Hispanic health disparities in ALL, while aberrations in the JAK/STAT pathway may be more relevant to health disparities unique to Hispanic Mexican-Americans. *KRAS* G12A and *STAT5B* N642H mutations in ALL are associated with relapse and may therefore be contributing to Hispanic health disparities in ALL. Furthermore, the JAK/STAT and chromatin remodeling pathways were found harboring mutations in our ALL borderland cohort. Given that these pathways are theoretically targetable, Tier X variants within these genes warrant investigation for driving leukemogenesis.

## Figures and Tables

**Table 1:** Patient information.

Patient	Diagnosis	Subtype	Status	Ethnicity
P1	ALL	T-ALL	Relapse	Hispanic
P2	ALL	pre-T-ALL	Relapse	Hispanic
P3	ALL	pre-T-ALL	New onset	Hispanic
P4	ALL	pre-T-ALL	New onset	Hispanic
P5	ALL	early pre-B-ALL	Relapse	Hispanic
P6	ALL	early pre-B-ALL	Relapse	Hispanic
P7	ALL	pre-T-ALL	Relapse	Hispanic
P8	ALL	early pre-B-ALL	New onset	Hispanic
P9	ALL	early pre-B-ALL	New onset	Hispanic

## Data Availability

Data generated in this study may be accessed from the UTEP Bioinformatics Repository (https://datarepo.bioinformatics.utep.edu/getdata?acc=B4U5YED0W0RIE3C) (accessed on 7 July 2021).

## Supplementary Materials

The following are available online at https://www.mdpi.com/article/10.3390/ijerph18147345/s1, Figure S1: Clinically relevant genes.
