# Supplementary material for: The Genomic Landscape of a Restricted ALL Cohort from Patients Residing on the U.S./Mexico Border
Source: Int J Environ Res Public Health. 2021 Jul 9;18(14):7345. doi: 10.3390/ijerph18147345 (PMC8307122; doi:10.3390/ijerph18147345)
Supplement: Supplementary file 1 [file ijerph-18-07345-s001.zip › ijerph-1187846-supplementary.pdf]

International Journal of
Environmental Research and Public Health

MDPI

Supplementary Material

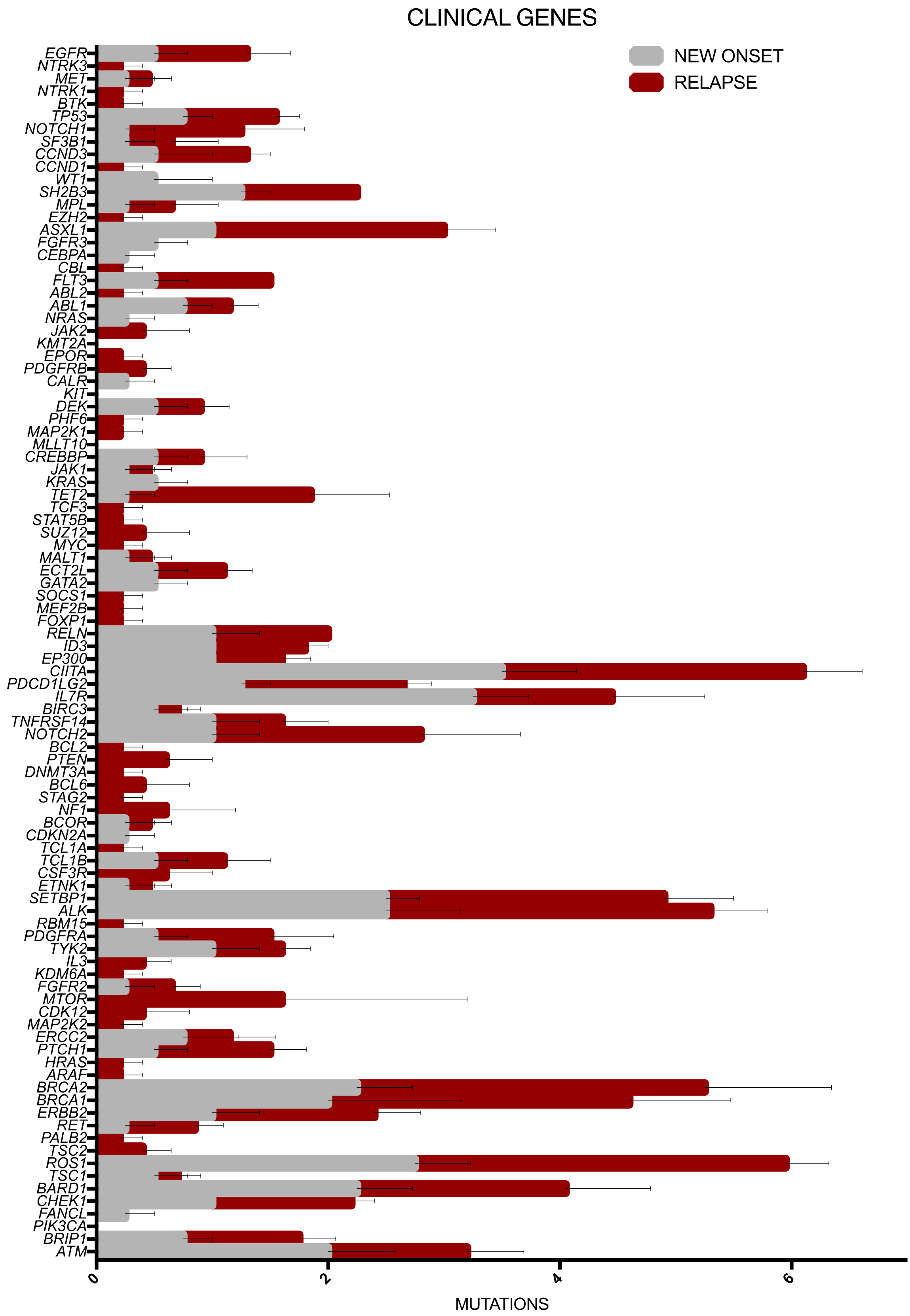

Figure S1. Clinically relevant genes. ALL mutations found in New onset (grey) and Relapse cases (maroon) are shown (Mean; SEM) in clinically relevant genes.
